# Profiles of viral pathogens from individuals with acute respiratory tract infections in northern Tanzania

**DOI:** 10.1016/j.ijregi.2025.100686

**Published:** 2025-06-13

**Authors:** Joshua Stephen Mollel, Shabani Ramadhani Mziray, Arnold Ndaro, Elimsaada Kituma, Innocent Kamwamwa, Lawrence Mapunda, Hadija Semvua, Stellah George Mpagama, Jaffu Othniel Chilongola

**Affiliations:** 1 School of Public Health, KCMC University, Moshi, Tanzania; 2 Department of Medical Biochemistry and Molecular Biology, KCMC University, Moshi, Tanzania; 3 Kibong’oto Infectious Diseases Hospital, Kilimanjaro, Tanzania; 4 Clinical Laboratory Department, Kilimanjaro Christian Medical Centre, Moshi, Tanzania; 5 National Public Health Laboratory, Dar es Salaam, Tanzania; 6 Faculty of Pharmacy, KCMC University, Moshi, Tanzania

**Keywords:** Acute respiratory tract infections, Viral pathogens, Influenza A virus, SARS-CoV-2, Human respiratory syncytial virus, PCR

## Abstract

**Objectives:**

Acute respiratory tract infections (ARTIs) may be misdiagnosed due to bottlenecks in conventional diagnostics. The primary objective was to identify viral pathogens using reverse transcription real-time polymerase chain reaction (RT-PCR).

**Methods:**

Cross-sectional study was conducted between 2022 and 2024 at Tanzanian referral hospitals. Viral RNA was extracted from oral-nasopharyngeal swabs and amplified using multiplex and singleplex RT-PCR. Cycle threshold values ≤40 and ≤35 were considered positive for multiplex and singleplex RT-PCR, respectively.

**Results:**

We recruited 183 participants with median age of 52 years (interquartile range, 32-69). Of these, 119 (65%) were females. The majority (167 of 183, 91.3%) had no COVID-19 vaccination. Over one-third (67 of 183, 36.61%) were PCR-positive for viral pathogens. Seven viral pathogens were identified, and the more prevalent were influenza A virus (Flu A) (n = 27), SARS-CoV-2 (n = 10), and respiratory syncytial virus (n = 7). The most common co-infecting viruses were FluA + respiratory syncytial virus (n = 6), Flu A + influenza B virus + SARS-CoV-2 (n = 4), and SARS-CoV-2 + Flu A (n = 3). Elderly people (aged >65 years) were commonly infected with Flu A (n = 9) and SARS-CoV-2 (n = 5).

**Conclusions:**

Viral pathogens are common in people with ARTIs. This study emphasizes consideration of RT-PCR in addition to conventional diagnostic approaches for identifying viral pathogens in patients with ARTIs.

## Highlights

• Influenza A virus is common in people with acute respiratory tract infection.
• Singleplex and multiplex polymerase chain reaction helps detection of respiratory viral pathogens.
• Elderly people are commonly infected with influenza A virus and SARS-CoV-2.
• Co-infecting viruses are common in people with acute respiratory tract infection.
• Hospitalization is associated with acute respiratory tract infections.

## Introduction

Acute respiratory tract infections (ARTIs), defined as self-limited irritation and swelling of the upper or lower respiratory pathways with associated cough, are caused by viruses, bacteria, or other pathogens [1]. The global burden of disease data from 1980 to 2016 indicates that acute ARTIs can progress to affect the upper and the lower respiratory system. Consequently, ARTIs have been linked to over 65 million hospital admissions across all age groups worldwide. Respiratory infections are among the top five leading causes of years of life lost and causing more than 56 million disability-adjusted life years worldwide. The impact of mortalities caused by lower respiratory tract infections is highly observed in low- to middle-income areas, especially in sub-Saharan Africa, South Asia, and Southeast Asia [2,3].

Most ARTIs are caused by viral infections, partly driven by, among other factors, risk factors that increase the incidence and severity of lower respiratory infection in low- to middle-income countries, including large family size, lateness in the birth order, crowding, low birth weight, malnutrition, vitamin A deficiency, lack of breast feeding, pollution, and young age [[4], [5], [6]]. The most frequently reported viral respiratory pathogens include influenza A (Flu A) and B viruses (Flu B), human respiratory syncytial virus (RSV), human parainfluenza viruses (HPIVs), adenovirus (HAdV), human rhinovirus, human metapneumovirus (HMPV), human coronaviruses, and SARS-CoV-2, which was responsible for the recent COVID-19 pandemic [[7], [8], [9], [10]]. The burden of viral ARTIs in East African region varies from 9% to 13%, with the highly prevalent viruses being HAdV (12%), RSV (10%), and HPIV (9%) [11].

The viral respiratory infections may be seasonal and present with non-specific overlapping symptoms such as cough, fever, headache, and difficulty in breathing [12]. This highlights the importance of accurately diagnosing the responsible pathogen because new pathogens may present similar symptoms. The severity of the symptoms in novel pathogens could be greater, particularly, in populations with an unprimed immune system. In Tanzania, access to laboratory diagnostics for respiratory viruses remains limited, particularly, in routine clinical settings, where such tools are not widely available for individual patient management. Treatment options, such as the use of oseltamivir, are not routinely accessible and generally reserved for severe cases or outbreak responses, often through vertical health programs or donor-supported initiatives.

Likewise, seasonal influenza vaccination is not part of the national immunization schedule and remains largely unavailable in the public health sector. These limitations emphasize the critical importance of integrating diagnostic tools within the broader public health surveillance efforts rather than solely focusing on individual patient care. Strengthening surveillance systems for respiratory viruses in resource-limited settings such as Tanzania is essential for policy development and timely public health interventions.

ARTI diagnosis has largely been syndromic without confirmation of the pathogens; this approach is unable to correctly detect respiratory pathogens linked to infections and inappropriate for reliably detecting new threats [13]. Occasionally, the application of conventional diagnostics such as serologic/immunologic tests may be used; however, there are widely reported limitations, including cross-reactivity, as there in seasonal coronaviruses vs SARS-CoV-2 [14]. In these scenarios, many cases of viral pathogens are misdiagnosed and further allow the spread of diseases and unfavorable treatment outcomes to patients. This study used reverse transcription real-time polymerase chain reaction (RT-PCR) to investigate viral pathogens in individuals presenting with ARTI at tertiary hospitals in northern Tanzania. The study explored the influence of COVID-19, the novel pathogen that emerged in 2019, and other traditional viral pathogens in ARTIs and explored associated factors.

## Materials and methods

### *Study design and setting*

This was a cross-sectional, hospital-based study conducted between January 2022 and April 2024. Data were collected from two referral hospitals in northern Tanzania: Kilimanjaro Christian Medical Centre (KCMC) and Mawenzi Regional Referral Hospital (Mawenzi RRH). KCMC is a university teaching hospital that serves as the zonal referral center for over 15 million people in northern Tanzania [15]. It provides care to approximately 15,000 patients each month and has an inpatient capacity of 634 beds, 100 of which are dedicated to the medical ward. The hospital experiences an average daily admission rate of 14 patients, with a bed occupancy rate of 99% and an average length of stay of 8 days. Mawenzi RRH, located in the center of Moshi town, is a 300-bed hospital serving the health care needs of the Kilimanjaro region, which has a population of approximately 1.86 million people according to the 2022 census. Mawenzi RRH handles over 300 out-patient visits daily. Both hospitals were selected due to their involvement in managing patients with COVID-19 during the pandemic. The climate of the northern part of Tanzania is characterized by a bimodal rainfall pattern: the “long rainy” season from March to May and the “short rainy” season from October to December, with a dry transition period from June to September [[16], [17], [18]].

### *Study population and sampling*

The study screened for symptomatic participants aged 2 months and older, presenting at the hospital with one or more ARTI symptoms/signs, namely, fever (axillary temperature >38°C), nasal congestion, sore throat, dry cough, tachypnea, purulent throat exudate, and cervical lymphadenopathy. Participants who consented (or whose parents/caregivers provided consent for minors) and had one or more of the mentioned signs/and symptoms were eligible for inclusion. The study excluded participants who did not allow nasopharyngeal swab collection. Likewise, those who had missing demographic and clinical data were excluded. A minimum sample size of 172 participants was estimated based on the methodology of a previous study [19]. Participants were selected through purposive and convenience sampling.

### *Data and sample collection procedures*

This study adopted the COVID-19 case investigation form version 5 from the Ministry of Health of Tanzania for comprehensive data collection. The form was structured to record key socio-demographic and clinical data from enrolled participants. Participants underwent clinical assessments by trained medical doctors who recorded medical history and performed physical examinations using the case investigation form. Nasopharyngeal swabs were collected aseptically, as previously described by Marty *et al*., [20] and placed into viral transport media (Capricorn Scientific GmbH, Germany). The oral pharyngeal swab was collected and mixed with a nasopharyngeal swab in the same tube. For minors, the procedure was performed as described by Pondaven-Letourmy *et al*. [21]. The collected samples were labeled and de-identified by assigning codes. The samples were immediately stored at −80°C for later laboratory examinations.

### *Laboratory procedures*

#### Aliquoting of frozen samples

For 1 hour, frozen samples were thawed gradually to prevent RNA degradation in the following sequence: −80°C to −40°C, −40°C to −20°C, then from −20°C to 4°C, before being placed on bench for downstream manipulations. After thawing, each sample was gently vortexed for 5 seconds to ensure homogeneity. The aliquoted samples were kept on ice and transported to Kibong'oto Infectious Disease Hospital for RNA extraction, amplification, and detection of SARS-CoV-2, RSV, Flu A, and Flu B. Subtyping of Flu A and detection of other non-influenza respiratory viruses were performed at the National Public Health Laboratory, Dar es Salaam.

#### Viral RNA extraction

SARS-CoV-2, Flu A, Flu B, and RSV RNA were extracted from 300 µL of nasopharyngeal and oropharyngeal samples using the Quick-DNA/RNA viral kit (Zymo Research Corporation, Irvine, CA, USA) following the manufacturer’s protocol. A total of 50 µL of purified viral RNA were eluted from spin columns using a relative centrifugal force of 16,000 × g for 30 seconds. The eluted viral RNA was immediately used for downstream amplification and pathogen detection. For the detection of other non-influenza viruses, viral RNA was extracted from 140 ul of the sample using the QIAamp Viral RNA Mini Kit (Qiagen, Germany) according to the manufacturer’s instructions. After adding 60 µl of Buffer AVE to each QIAamp Mini column, the samples were incubated at room temperature for 1 minute and then centrifuged at 16,000 × g for 1 minute. The eluted RNA was immediately used for amplification and detection of non-influenza respiratory viruses.

### *Amplification and detection of SARS-CoV-2, Flu A, Flu B, and RSV*

Preparations for amplification and detection of the four viral targets, namely, SARS-CoV-2, FluA, FluB, and RSV, was done using Allplex SARS-CoV-2/FluA/FluB/RSV RT-PCR assay kit (Seegene Inc, Seoul, Republic of Korea). RT-PCR was conducted using the Bio-Rad CFX96 Real-Time PCR Detection System (Hercules, CA, USA). Briefly, for each reaction, a master mix was prepared by combining equal volumes (5 µl) of probes/primer (SC2FabR MOM) and reverse transcriptase enzyme (EM8), followed by brief vortexing. Then, 10 µl of the master mix was aliquoted into polymerase chain reaction (PCR) tubes, to which 10 µL of the extracted nucleic acids from each sample were added, along with 10 µl of a positive control (SC2FabR PC) and 10 µl of RNase-free water as a negative control. The PCR tubes were briefly centrifuged and then loaded into the RT-PCR detection system. Reverse transcription and inactivation of the reverse transcriptase was done at 50°C for 20 minutes and 95°C for 15 minutes, respectively. Subsequently, three cycles of denaturation at 95°C for 10 seconds, annealing at 60°C for 40 seconds and extension at 72°C for 20 seconds, were carried out. This was followed by 41 cycles of denaturation at 95°C for 10 seconds, with fluorescence detection at 60°C for 15 seconds and 72°C for 20 seconds. All runs were performed alongside relevant controls to ensure data validity. PCR results were analyzed using Seegene Viewer v2.0 (Seegene Inc, Seoul, South Korea), where samples with cycle threshold (Ct) values ≤40 were considered positive. Any remaining clinical samples and residual nucleic acid extracts were stored at −80°C for future analysis.

### *Subtyping of influenza A virus*

For all Flu A PCR–positive samples, additional targeted amplification and detection was performed to characterize their subtypes using the Centers for Disease Control and Prevention (CDC) RT-qPCR Flu A virus (H3, H1pdm09) subtyping panel. Briefly, 20 µl of RT-PCR master mix containing buffer, forward and reverse primers, probes, reverse transcriptase, and nuclease-free water was put into 96-well PCR plates. A total of 5 µl of PCR-positive Flu A viral RNA extract was added to each well, along with positive control, negative control (nuclease-free water), and human sample control. Amplification was carried out using the Bio-Rad CFX96 Dx system (Hercules, CA, USA). Thermal cycling conditions were as follows: pre-heating and reverse transcription at 20°C for 2 minutes and 50°C for 15 minutes, respectively, initial denaturation/inactivation of reverse transcriptase at 95°C for 3 minutes, followed by 45 cycles of denaturation at 95°C for 15 seconds, and annealing/extension at 55°C for 30 seconds. The results were interpreted according to CDC guidelines, where a Ct value ≤35 was considered positive for the presence of Flu A virus or its subtypes.

### *Amplification and detection of other non-influenza respiratory viruses*

In addition to the Allplex PCR kit, we used the US CDC’s respiratory RNA virus panel of singleplex reverse transcription RT-PCR to determine six other viruses, namely, HMPV; HPIV 1, 2, and 3; HAdV; and human rhinovirus. All the necessary reagents were made in the AgPath-ID one-step RT-PCR kit (Applied Biosystems, TX, USA). Briefly, a reaction mix was prepared by combining 5 µl of viral RNA with 20 µl of master mix, which included 12.5 µl of 2× RT-PCR buffer, 0.5 µl of each primer (20 µM), 0.5 µl of TaqMan probes (6 µM), 1 µl of 25× RT-PCR enzyme mix (SuperScript III One-Step RT-PCR System with Platinum Taq DNA Polymerase, Life Technologies), and 5 µl of nuclease-free water. After vortexing the master mix briefly, 10 µl was aliquoted into each PCR tube. The reaction mix was sealed and centrifuged briefly before RT-PCR amplification. Thermal cycling involved reverse transcription at 45°C for 10 minutes, followed by initial denaturation/inactivation of reverse transcriptase at 95°C for 10 minutes. The amplification phase consisted of 45 cycles of denaturation at 95°C for 15 seconds and annealing/extension at 55°C for 1 minute. Samples with Ct ≤35 were considered positive for the targeted respiratory viruses. The PCR was performed on Bio-Rad CFX96 Real-Time PCR Detection System (Hercules, CA, USA).

### *Statistical analysis*

Microsoft Excel was applied to visualize and clean missing data. Clean data were exported to and analyzed using STATA version 15.0. Continuous variables were summarized using median and interquartile range due to non-normal distribution, whereas categorical variables were summarized using frequencies and proportions. The initial analysis of predictors of viral infections was done using Fisher’s exact test and Chi-Square test, with a significance level of *P* <0.05. Additional analysis of predictors of viral infection was performed using multivariable logistic regression whereby the strength of association was evaluated by odds ratios (ORs) and 95% confidence interval.

## Results

### *Characteristics of study participants*

A total of 183 participants met the study inclusion criteria and provided metadata and samples for downstream laboratory examinations. Of these, 119 (65%) were females. The median age of participants was 52 years (interquartile range, 32-69) and over one-third of them (62 of 183, 33.9%) were elderly people aged above 65 years. Nearly half of the participants (90 of 183, 49.2%) were peasants, and almost all (177 of 183, 96.7%) had no history of traveling outside the country (Table 1). Over two-thirds of participants (126 of 183, 68.85%) were out-patients, whereas 57 (31.2%) were in-patients. Majority of the participants (167 of 183, 91.3%) had not received COVID-19 vaccination (Table 2).

**Table 1:** Socio-demographic characteristics of participants (N = 183).

Characteristic	Frequency	Percentage^a^
**Age in years**		
≤5	5	2.7
6-15	6	3.3
16-25	13	7.1
26-35	27	14.8
36-45	27	14.8
46-55	20	10.9
56-65	23	12.6
>65	62	33.9
Median age = 52, interquartile range (32-69)		
**Occupation**		
Health care workers	25	13.7
Business	14	7.7
Peasants	90	49.2
Other	19	10.4
Children/Students	35	19.1
**Hospital attended**		
Mawenzi RRH	79	43.2
KCMC	104	56.8
**Sex**		
Female	119	65.0
Male	64	35.0
**History of travel outside the country**		
No	177	96.7
Yes	6	3.3

KCMC = Kilimanjaro Christian Medical Centre Zonal Referral Hospital; Mawenzi RRH = Mawenzi Regional Referral Hospital.

a Percentages were computed using 183 as the denominator.

**Table 2:** Clinical characteristics of participants (N = 183).

Characteristic	Frequency	Percentage^a^
**Type of patient**		
Out-patient	126	68.8
In-patient	57	31.2
**Duration of illness (days)**		
1-7	96	52.5
>7	87	47.5
Median days = 7, interquartile range (3-14)		
**COVID-19 vaccination**		
No	167	91.3
Yes	16	8.7
**Comorbidities**		
No	119	65.0
Yes	64	35.0
**Fever/Chills**		
No	128	69.9
Yes	55	30.1
**Nausea/Vomiting**		
No	100	55.0
Yes	83	45.0
**Cough**		
No	97	53.0
Yes	86	47.0
**Difficulty in breathing**		
No	50	27.3
Yes	133	72.7
**Chest pain**		
No	126	68.8
Yes	57	31.2
**Body weakness**		
No	96	52.5
Yes	87	47.5
**Sore throat**		
No	105	57.4
Yes	78	42.6
**Loss of smell/taste**		
No	120	65.6
Yes	63	34.4
**Runny nose**		
No	160	87.0
Yes	23	13.0

a Percentages were computed using 183 as the denominator.

### *Profiles of respiratory viral infections and co-infections*

**Figure 1:**
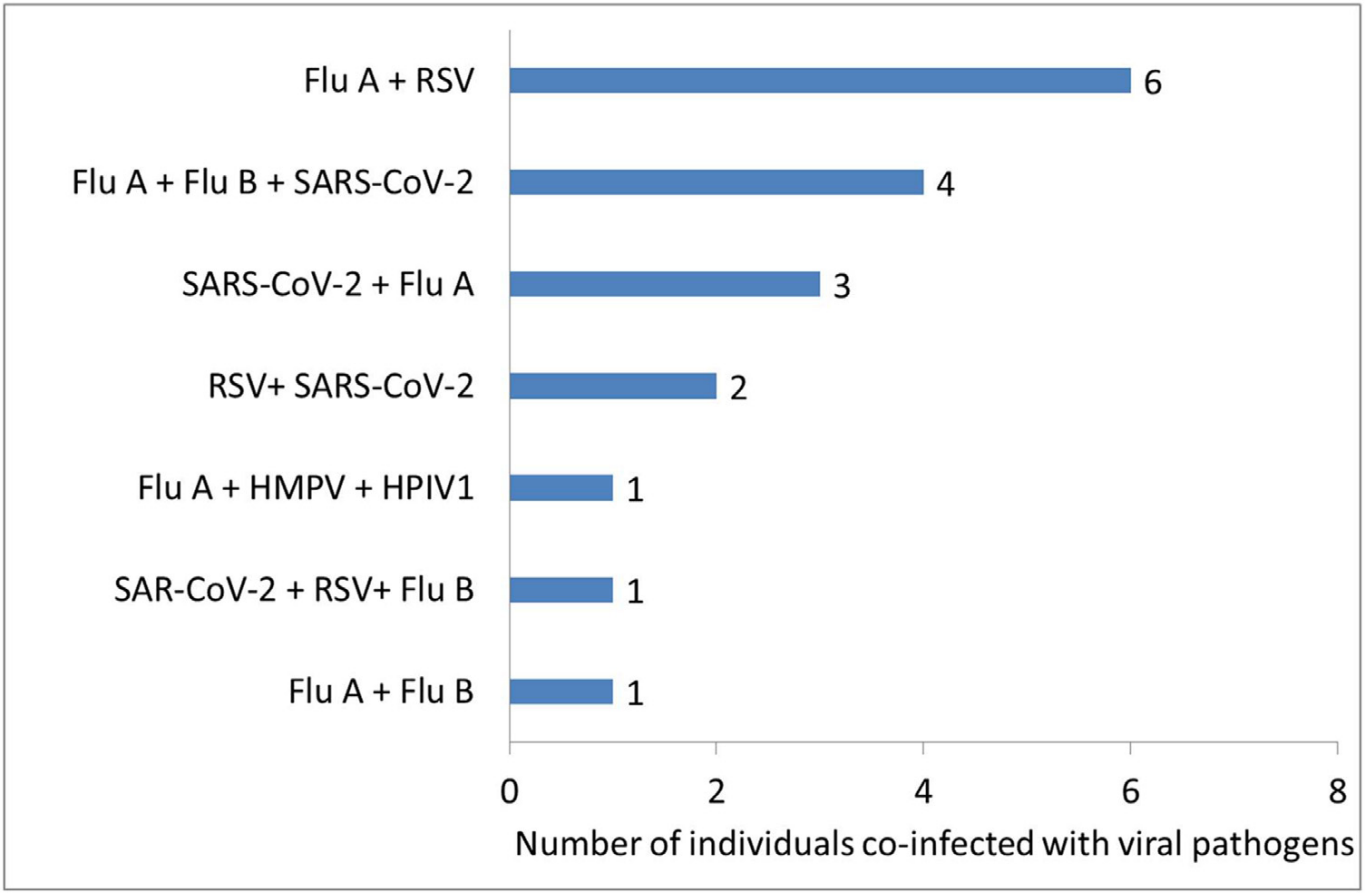
Description of dual and triple viral co-infections (n = 18) in individuals presenting with acute respiratory infections. The figure presents four dual co-infections such as Flu A + RSV, SARS-CoV-2 + Flu A, RSV+ SARS-CoV-2, and Flu A + Flu B. Triple co-infections include Flu A+ Flu B+ SARS-CoV-2, Flu A + HMPV + HPIV1, and SARS-CoV-2 + RSV + Flu B. HADV, human adenovirus; HMPV, human metapneumovirus; HPIV1, human parainfluenza virus type 1; Flu A, influenza A virus; Flu B, influenza B virus; RSV, respiratory syncytial virus.

Of 183 samples from individuals with ARTI, 67 (36.61%) were PCR-positive for viral infections. Seven viral pathogens were identified, and the most prevalent were Flu A (n = 27), SARS-CoV-2 (n = 10), and RSV (n = 7) (Table 3). Other detected viruses included HMPV (n = 3), HAdV (n = 1), HPIV1 (n = 1) (Table 3), and Flu B, which appeared in co-infection (n = 6) (Figure 1). All the Flu A viruses were subtype H3, which causes seasonal flu in humans. We observed co-infecting viruses in 18 individuals and the most common were FluA + RSV (n = 6), Flu A + Flu B + SARS-CoV-2 (n = 4), and SARS-CoV-2 + Flu A (n = 3). Figure 1 further describes other co-infecting viral pathogens. Flu A was the only virus detected in all age groups (Table 3). The age distribution of individuals with respiratory viral co-infections further showed that co-infections were most prevalent among adults aged 26-35 years, accounting for 33.3% of all co-infected cases, followed by those aged 36-45 years (27.9%). Elderly individuals over 65 years also had a prevalence of 16.6% of co-infections, alongside those aged 56-65 years (16.6%) and were commonly infected with Flu A (n = 9) and SARS-CoV-2 (n = 5). In contrast, co-infections were rare among children under 5 years (5.6%) and absent in the 6-25 and 46-55 years age groups (Table 3).

**Table 3:** Age-disaggregated distribution of viral pathogens among individuals with ARTIs, N = 67.

Age in years	Proportions of detected respiratory viruses							**Total**^a^
	RSV n (%)	Flu A n (%)	SARS-CoV-2 n (%)	HADV n (%)	HMPV n (%)	HPIV1 n (%)	Co-infection n (%)	
≤5	0 (0)	1(3.7)	0(0)	0(0)	0(0)	0(0)	1(5.6)	2
6-15	1(14.3)	2(7.4)	0(0)	0(0)	0(0)	0(0)	0(0)	3
16-25	0 (0)	2(7.4)	0(0)	0(0)	0(0)	0(0)	0(0)	2
26-35	0(0)	2(7.4)	1(10)	1(100)	0(0)	0(0)	6(33.3)	10
36-45	1(14.3)	3(11.1)	2(20)	0(0)	2(66.7)	0(0)	5(27.9)	13
46-55	2(28.6)	6(22.2)	0(0)	0(0)	1(33.3)	0(0)	0(0)	9
56-65	1(14.3)	2(7.4)	2(20)	0(0)	0(0)	0(0)	3(16.6)	8
>65	2(28.5)	9(33.4)	5(50)	0(0)	0(0)	1(100)	3(16.6)	20
Total	7	27	10	1	3	1	18	**67**

HADV, human adenovirus; HMPV, human metapneumovirus; HPIV1, human parainfluenza virus type 1; Flu A, influenza A virus; RSV, respiratory syncytial virus.

a Total number of individuals infected with viruses per age category.

### *Predictors of viral respiratory tract infections*

Of 67 individuals with PCR-positive samples for viral infections, nearly one-third (20 of 67, 29.9%) were elderly people aged above 65 years. In addition, among the people with viral infections, 59 of 67 (88%) were attending out-patient department and the most common symptoms presented were difficulty in breathing, sore throat, loss of smell, and nausea (Table 4). Participants who were hospitalized had 5.4 times higher odds of having respiratory tract viral infections than those attending the out-patient department (crude OR = 5.4, 95% CI [2.4-12.3]). However, although not statistically significant at crude analysis, participants who had comorbidities had 1.8 higher odds of having respiratory tract viral infections than those who had no comorbidities (crude OR = 1.8, 95% CI [0.9-3.4]) (Table 4). Table 4 further presents multivariable logistic regression results that only hospitalization was an independent predictor of respiratory tract viral infections (adjusted OR = 5.2, 95% CI [2.2-12.3]).

**Table 4:** Predictors of respiratory tract viral infection (N = 183).

Characteristic	Viral infection			Crude analysis	Multivariable analysis
	Detected = 67 n (%)	Not detected = 116 n (%)	*P*-value	Crude OR (95% CI)	Adjusted OR (95% CI)
**Median age in years [**interquartile range**]**	51 [32-66]	55 [33-70]	0.860	1 (0.98-1.02)	-
**Sex**					
Female	42 (62.7)	77 (66.4)	0.614	1	-
Male	25 (37.3)	39 (33.6)		1.17 (0.6-2.2)	
**Travel history**					
No	64 (96.0)	113 (97.0)	0.385*	1	-
Yes	3 (4.0)	3 (3.0)		0.6 (0.1-2.9)	
**Type of patient**					
Out-patient	59 (88.0)	67 (58.0)	<0.001	1	1
In-patient	8 (12.0)	49 (42.0)		5.4 (2.4-12.3)	5.2 (2.2-12.3)
**Duration of illness (days)**					
1-7	40 (59.7)	56 (48.3)	0.097	1	-
>7	27 (40.3)	60 (51.7)		1.6 (0.9-2.9)	
**COVID-19 Vaccination**					
No	64 (95.5)	103 (88.8)	0.906*	0.4 (0.1-1.4)	-
Yes	3 (4.5)	13 (11.2)		1	
**Comorbidities**					
No	52 (77.6)	73 (62.9)	0.040	1	1
Yes	15 (22.4)	43 (37.1)		1.8 (0.9-3.4)	1 (0.5-2.3)
**Fever/Chills**					
No	57 (85.1)	71 (61.2)	0.001	-	-
Yes	10 (14.9)	45 (38.8)			
**Nausea/Vomiting**					
No	26 (38.8)	74 (63.8)	<0.001	-	-
Yes	41 (61.2)	42 (36.2)			
**Cough**					
No	47 (70.2)	50 (43.1)	<0.001	-	-
Yes	20 (29.8)	66 (56.9)			
**Difficulty in breathing**					
No	15 (22.4)	35 (30.2)	0.255	-	-
Yes	52 (77.6)	81 (69.8)			
**Chest pain**					
No	54 (80.6)	72 (62.1)	0.009	-	-
Yes	13 (19.4)	44 (37.9)			
**Body weakness**					
No	33 (49.3)	63 (54.3)	0.509	-	
Yes	34 (50.7)	53 (45.7)			
**Sore throat**					
No	24 (35.8)	81 (69.8)	<0.001	-	-
Yes	43 (64.2)	35 (30.2)			
**Loss of smell/taste**					
No	29 (43.3)	91 (78.5)	0.001	-	-
Yes	38 (56.7)	25 (21.5)			
**Runny nose**					
No	52 (77.6)	108 (93.1)	0.002	-	-
Yes	15 (22.4)	8 (6.9)			

CI, confidence interval; OR, odds ratio.

*P-value computed using Fisher's Exact test.

## Discussion

Our study revealed that singleplex and multiplex RT-PCR testing identified multiple viral pathogens in more than one-third (36.61%) of patients with ARTIs at referral hospitals in northern Tanzania. The common viral pathogens identified in this population were Flu A subtype H3, which causes seasonal flu in humans, SARS-CoV-2, and human RSV. Other viruses include HMPV, HAdV, HPIV1, and Flu B. Our findings mirror the previous results from studies done in the national sentinel surveillance system for influenza in pre–COVID-19 pandemic era and confirm circulation of the respiratory viruses in Tanzania [10,22]. Although the World Health Organization declared that SARS-CoV-2 is no longer a pathogen of pandemic priority, its threat to global health remains [23]. Thus, conducting surveys or surveillance of ARTIs, including SARS-CoV-2, warrant strengthening of infection prevention and control strategies to minimize potential epidemics and pandemics. In addition, we advocate continued surveillance of respiratory virus pathogens using expanded panels of PCR test kits. Our argument is corroborated by the fact that we applied two PCR assays, namely, Allplex SARS-CoV-2/Flu A/Flu B/RSV assay, to detect four viruses concurrently and the CDC-expanded respiratory virus RT-PCR assay to attempt detection of six more respiratory viruses. This approach enhanced the detection capacity, allowing confirmation of seven viruses and their combinations, as described previously. However, due to increased antigenic shift, drift, and genetic diversity of respiratory viruses such as Flu A, novel strains resulting from these changes could be missed by PCR. Although this study did not use metagenomics next-generation sequencing, its unbiased approach could greatly enhance the surveillance and detection of novel viral strains [24]. Routine surveillance of the respiratory viral pathogens is equally recommended to be strengthened in the animal health sector as well because some of these viruses are zoonotic, as was recently reported in the United States where Flu A subtype H3 infected pigs [25].

We observed dual and triple co-infecting viruses in 18 individuals and the most common were FluA + RSV, Flu A + Flu B + SARS-CoV-2, and SARS-CoV-2 + Flu A. Co-infections, particularly, those involving Flu A, SARS-CoV-2, and RSV, are a notable concern in the management of patients with ARTI. They have been linked to more severe clinical outcomes, potentially due to the complex host immune interactions with different pathogens. Multiple infections may cause more immune system disruption, leading to more severe symptoms or prolonged illness [12]. The increased notification of viral co-infections in adults aged 26-45 years in this study suggests that this age group may be vulnerable to viral co-infections. This could be due to increased exposure in community and occupational settings, where individuals are more likely to encounter multiple pathogens [26].

The study further reports that elderly people with more than 65 years of age are commonly infected with Flu A and SARS-CoV-2. This finding is in line with previous reports which suggest that older individuals may have a greater susceptibility to viral infections, which could be due to age-related immune decline, increased vulnerability to pathogens, and higher exposure to health care settings [27]. It was interesting, however, to report a lower detection rate of respiratory viruses, especially RSV in children than in adults, despite the general understanding that children are typically at higher risk of exposure to viral respiratory infections due to many factors, including weakened immune systems [28]. This could be related to the relatively small sample size of children in the study or possibly due to a cohort effect, where the higher burden of disease is seen in older age groups who may have accumulated a greater number of exposure opportunities to viruses over time.

Hospitalization was the only independent predictor of respiratory tract viral infections in this study. However, due to the lack of data on the timing of symptom onset, admission, and testing, it is not possible to establish whether this association reflects nosocomial transmission. Nonetheless, this finding underscores the critical importance of strengthening infection prevention and control measures in health care settings. These measures should include the isolation of confirmed cases of highly infectious diseases and the segregation of patients where feasible [29,30]. Similarly, in out-patient departments, improving the identification and separation of patients with suspected or probable ARTIs remains essential to reducing the potential risk of transmission.

## Conclusion and recommendation

Viral pathogens are common in individuals presenting with ARTIs. This study underscores the importance of considering the application of advanced molecular diagnostic tools, such as multiplex PCR in addition to existing conventional diagnostic technologies, for accurate identification of viral pathogens from patients with ARTIs. Although clinical symptoms can guide suspicion of viral infections, they are insufficient on their own to confirm diagnosis. The study highlights the importance of using PCR assays to support clinical diagnosis by accurate confirmation of viral pathogens from patients with ARTIs. We equally advocate for expanded panels of PCR assays for enhanced surveillance of influenza and non-influenza respiratory viruses and guide effective public health interventions and improve health outcomes. Given the high prevalence of Flu A detected during the study period, the introduction and broader implementation of influenza vaccination, particularly, among high-risk groups such as young children, elderly individuals, and individuals with chronic health conditions, could offer significant public health benefits. Strengthening access to seasonal influenza vaccines within the Tanzanian health care system should be considered as part of broader efforts to reduce the burden of respiratory infections.

## Declarations of competing interest

The authors have no competing interests to declare.
