# Corrigendum to ‘Profiles of viral pathogens from individuals with acute respiratory tract infections in northern Tanzania’ [IJID Regions 16 (2025) 100686]

**DOI:** 10.1016/j.ijregi.2026.100853

**Published:** 2026-02-07

**Authors:** Joshua Stephen Mollel, Shabani Ramadhani Mziray, Arnold Ndaro, Elimsaada Kituma, Innocent Kamwamwa, Lawrence Mapunda, Hadija Semvua, Stellah George Mpagama, Jaffu Othniel Chilongola

**Affiliations:** 1 School of Public Health, KCMC University, Moshi, Tanzania; 2 Department of Medical Biochemistry and Molecular Biology, KCMC University, Moshi, Tanzania; 3 Kibong’oto Infectious Diseases Hospital, Kilimanjaro, Tanzania; 4 Clinical Laboratory Department, Kilimanjaro Christian Medical Centre, Moshi, Tanzania; 5 National Public Health Laboratory, Dar es Salaam, Tanzania; 6 Faculty of Pharmacy, KCMC University, Moshi, Tanzania

The authors regret that the funding acknowledgement in the above published article was not correct.

Instead of “This work was supported by EACCR3 through the Neglected Infectious Diseases Work Package-5 at Kilimanjaro Clinical Research Institute. It was also partly funded by the EDCTP2 program supported by the European Union project (grant number TMA2016SF-1463-REMODELTZ)”, it should have stated “This work was supported by EACCR3 through the Neglected Infectious Diseases Work Package-5 at Kilimanjaro Clinical Research Institute. EACCR3 is part of the EDCTP2 programme supported by European Union under grant agreement CSA 2020NOE-3102. It was also partly funded by the EDCTP2 program supported by the European Union project (grant number TMA2016SF-1463-REMODELTZ)”.

Also, in the affiliation section, it was mistakenly written as “Faculty of Pharmacy” instead of “School of Pharmacy.”

The authors would like to apologise for any inconvenience caused.

